# Two outer membrane proteins are bovine lactoferrin-binding proteins in *Mannheimia haemolytica* A1

**DOI:** 10.1186/s13567-016-0378-1

**Published:** 2016-09-06

**Authors:** Luisa Samaniego-Barrón, Sarahí Luna-Castro, Carolina Piña-Vázquez, Francisco Suárez-Güemes, Mireya de la Garza

**Affiliations:** 1 Departamento de Biología Celular, Centro de Investigación y de Estudios Avanzados del IPN (CINVESTAV-IPN), Avenida Instituto Politécnico Nacional No. 2508, Colonia San Pedro Zacatenco, CP 07360 Ciudad de México, Mexico; 2 Facultad de Medicina Veterinaria y Zootecnia Dr. Norberto Treviño Zapata, Universidad Autónoma de Tamaulipas, Carretera a Cd. Mante Km 5, CP 87000 Ciudad Victoria, Tamaulipas Mexico; 3 Departamento de Microbiología, Facultad de Medicina Veterinaria y Zootecnia, Universidad Nacional Autónoma de México (UNAM), Av. Universidad 3000, Cd. Universitaria, Coyoacán, CP 04510 Ciudad de México, Mexico

## Abstract

*Mannheimia haemolytica* is a Gram negative bacterium that is part of the bovine respiratory disease, which causes important economic losses in the livestock industry. In the present work, the interaction between *M. haemolytica* A1 and bovine lactoferrin (BLf) was studied. This iron-chelating glycoprotein is part of the mammalian innate-immune system and is present in milk and mucosal secretions; Lf is also contained in neutrophils secondary granules, which release this glycoprotein at infection sites. It was evidenced that *M. haemolytica* was not able to use iron-charged BLf (BholoLf) as a sole iron source; nevertheless, iron-lacked BLf (BapoLf) showed a bactericidal effect against *M. haemolytica* with MIC of 4.88 ± 1.88 and 7.31 ± 1.62 μM for *M. haemolytica* strain F (field isolate) and *M. haemolytica* strain R (reference strain), respectively. Through overlay assays and 2-D electrophoresis, two OMP of 32.9 and 34.2 kDa with estimated IP of 8.18 and 9.35, respectively, were observed to bind both BapoLf and BholoLf; these OMP were identified by Maldi-Tof as OmpA (heat-modifiable OMP) and a membrane protein (porin). These *M. haemolytica* BLf binding proteins could be interacting in vivo with both forms of BLf depending on the iron state of the bovine.

**Electronic supplementary material:**

The online version of this article (doi:10.1186/s13567-016-0378-1) contains supplementary material, which is available to authorized users.

## Introduction

*Mannheimia haemolytica* is an opportunistic Gram-negative bacterium that belongs to the *Pasteurellaceae* family. This bacterium is part of the bovine respiratory disease (BRD), which causes important economic losses in livestock. The influence of the environment, stressing factors, and infections by viruses and bacteria, causes BRD; these factors seem to alter the bovine upper respiratory-tract epithelium allowing *M. haemolytica* to colonize it, escape clearance and move from the nasopharynx to the lungs, leading to pneumonia [1]. Transportation of animals is the most accepted non-infectious risk factor including distance, method, and dehydration; ambient factors, like abrupt and extreme changes in weather conditions, rather than simply cold or inclement weather, predispose cattle to BRD. Among bovine viruses, the main are herpes-1, respiratory syncytial, viral diarrhea, and parainfluenza-3, all of them causing immunosuppression [2–4]. The bacterial pathogens that more frequently infect after the viral infection are *M. haemolytica* and *Pasteurella multocida*. Other pathogens are *Histophilus somni*, *Mycoplasma bovis*, and *Trueperella pyogenes.* There are twelve *M. haemolytica* serotypes, from which, A1 is found with more frequency in pneumonic bovines [5]. *M*. *haemolytica* infection causes a fibrinosuppurative and necrotizing inflammatory response in the lungs. *M*. *haemolytica* possesses numerous pathogenicity mechanisms that allow bacterial invasion and colonization leading to pulmonary injury. Within the reported pathogenicity, determinants are leukotoxin [6]; lipopolysaccharide (LPS) [7]; capsule [8]; outer membrane proteins (OMP) and membrane lipoproteins [9–11]; adhesins [12]; fimbriae [13]; enzymes (neuraminidase, metalloglycoproteases, and IgA and IgG proteases) [14]; and antimicrobial resistance plasmids [15–17]. *M*. *haemolytica* possesses specific iron uptake mechanisms for bovine holotransferrin (BholoTf) (TbpA and TbpB of 71 and 77 kDa, respectively, in the A1 serotype) [18]. Interestingly, *M. haemolytica* does not produce siderophores; however, receptors for siderophores have been found in its genome. *M*. *haemolytica* genes coding HmbR1 and HmbR2 proteins to capture hemoglobin and the use of this ferrous protein as an iron source have been demonstrated [19].

Lactoferrin (Lf) is an 80 kDa cationic non-heme glycoprotein that belongs to the mammalian innate-immune system, with higher affinity for iron than transferrin (Tf); depending on the amount of iron, Lf could be iron-free (apoLf) or charged with one or two iron atoms (holoLf) [20]. Lf is found in high concentration in colostrum and milk (5–7 and 1 mg/mL, respectively), and in a lower concentration in other body secretions, such as intestinal and respiratory secretions. Lf is also produced by the secondary granules of neutrophils, which release this protein at infection sites [21]. Lf is a multifunctional protein, since it displays serine protease activity, and it is also antiinflammatory, immunomodulatory, and anticarcinogenic [22]. The interaction of Lf with microorganisms leads to different outcomes depending on the Lf form; in this sense, holoLf can serve as an iron source whereas apoLf generally causes microbial death. ApoLf can be bacteriostatic, since it chelates the iron needed for pathogenic growth in fluids and mucosae. In addition, apoLf can cause death in certain pathogenic species, because Lf alters the OM permeability causing the release of LPS in Gram negative bacteria. Lf also binds to porins affecting their permeability and thus allowing the influx of toxins and antibiotics [23–26]. In addition, it has been demonstrated that the N-terminus of Lf is responsible for the bactericidal effect; peptides from this N-terminus can be obtained by cleavage with pepsin, and they have been named lactoferricins (Lfcins) [27]. Lf also inhibits bacterial aggregates and biofilm formation. Interestingly, apoLf can potentiate the bactericidal effect of antimicrobials; our group demonstrated *A. pleuropneumoniae* death by apoLf, and a synergistic activity of apoLf with oxytetracycline [28]. The bactericidal effect of apoLf has also been demonstrated in *Aggregatibacter actinomycetemcomitans* [29]; both bacterial species belong to the *Pasteurellaceae* family. Concerning the use of holoLf as a sole iron source, the iron uptake by receptors has been more extensively studied in the *Neisseriaceae* family [30]. In *Neisseria meningitidis, Neisseria gonorrhoeae*, *Moraxella catarrhalis* and *Moraxella bovis*, the OMP LbpA and LbpB (105 and 80–100 kDa, respectively) have been described as binding proteins to holoLf. In addition, Lbps of 105 and 106 kDa displayed binding to human holoLf in *Haemophilus influenzae*, another member of the *Pasteurellaceae* family [31]. However, *lbpA* and *lbpB* genes homologous to those of the *Neisseriaceae* family were not found in the genomes of *A. pleuropneumoniae* and *M. haemolytica* [18]. Since *M. haemolytica* is constantly interacting with the bovine innate-immune system in respiratory mucosa, the aim of this work was to determine the type of relationship that takes place between the host Lf and *M. haemolytica.* A bactericidal effect was found for BapoLf, meanwhile holoLf was not used by the bacteria as a sole iron source. Both apoLf and holoLf were bound to two OMP of 32.9 and 34.2 kDa with estimated IP of 8.18 and 9.35, which were identified as OmpA (heat-modifiable protein) and a membrane protein (porin), respectively.

## Materials and methods

### Strains and growth conditions

Two strains of *M. haemolytica* A1 were used, a field isolate from a pneumonic bovine (MhF), previously identified by conventional culture and API 20E tests, as well as indirect hemagglutination assay to establish the serotype [5]. The other strain (MhR) was kindly donated by G. H. Frank and R. E. Briggs from the National Animal Disease Center, United States Department of Agriculture. *Actinobacillus pleuropneumoniae* serotype 1 (strain S4074) was used as a negative control of BholoLf utilization as an iron source [28]; this strain was kindly donated by M. Gottschalk (Groupe de Recherche sur Maladies Infectieuses de Porc, Université de Montréal, Canada). An isolate of *Moraxella bovis* from a bovine suffering keratoconjunctivitis (identified by API 20E test), was used as a positive control of BholoLf utilization as an iron source [32]. *Mannheimia haemolytica* and *M. bovis* strains were regularly grown in 5% sheep blood agar for their use. *A*. *pleuropneumoniae* was regularly grown in TSA plus NAD (15 µg/mL).

### Reagents

Bovine apoLf (BapoLf) was purchased at NutriScience Innovations LLC, USA, and contained 0.005% iron. BapoLf was saturated with iron to obtain BholoLf according to the method described by Schryvers and Morris [33]; iron in BholoLf was 91.6%; it was quantified by an enzymatic automated method (MicroTech Laboratories, Mexico). Lactoferricin B (fragment 4–14), 2,2’-dipyridyl and bovine serum albumin (BSA) were purchased from Sigma. The protein assay for method of Bradford, was purchased from Bio-Rad, USA.

### Use of bovine hololactoferrin as a sole iron source by *M. haemolytica* A1

First, several concentrations (0.1–0.5 mM) of the iron-chelating agent 2′2 dipyridyl were added to BHI broth (Dibico, México), to determine an optimal iron chelation without causing bacterial death. After that, a minimal ferric-iron concentration was established for *M. haemolytica* growth by testing concentrations of 20, 40, 60, 80 and 100 μM FeCl_3_ in the presence of 0.4 mM 2,2’-dipyridyl. The optimal 2′2 dipyridyl concentration for *M. bovis* and *A. pleuropneumoniae* were 0.15 and 0.5 mM, respectively. Later, to know whether *M. haemolytica* can use BholoLf as an iron source, an initial culture in BHI broth at an OD_595_ = 0.02 was subcultured in different conditions: (1) BHI broth as a positive control of growth; (2) iron-chelated broth as a negative control of growth; (3) iron-chelated broth with 80 μM FeCl_3_; (4) iron-chelated broth supplemented with BholoLf (80 μM iron concentration). *A*. *pleuropneumoniae* serotype 1 and *M. bovis* were used as negative and positive control of BholoLf utilization as an iron source, respectively. Samples were incubated at 37 °C with agitation (200 rpm) and the OD_595 nm_ was registered at 24 h. Growth was done in three independent experiments each in triplicate. The results were expressed as the mean ± standard deviation and the statistical significance was searched with the Student’s *t* test.

### Effect of bovine apolactoferrin on the growth of *M. haemolytica*

The minimum inhibitory concentration (MIC) of BapoLf on the *M. haemolytica* A1 growth was determined employing the method of microdilution in BHI broth plus BapoLf. Bacteria (10^5^ UFC) were incubated with 0, 3.25, 6.5, 9.75, 13.0, 16.25, 19.5, 22.75 and 26 μM BapoLf up to 18 h at 37 °C in sterilized 96 well plates, and the OD at 595 nm was recorded. All of the experiments were repeated three times in triplicate.

### OMP extraction and overlay

Overlays were performed from *M. haemolytica* OMP. First, OMP were extracted according to the protocol previously described by Brennan [34]. Bacteria were harvested from BHI broth by centrifugation at 6000 × *g*, washed twice in 20 mM Tris (pH 7.2), and sonicated with 0.75″ probe (55 μm, amplitude setting 9.20 Hz) on ice for 10 min. Sonicate was centrifuged at 6000 × *g* for 20 min to remove cell detritus. The supernatant was removed with a pipette, placed into a clean tube and pelleted at 60 000 × *g* for 1 h. The supernatant was then removed and the pellet was suspended in 1 mL of 20 mM Tris (pH 7.2). Cytoplasmic membranes were solubilized by adding 4 mL of 0.5% *N*-lauryl-sarcosine and incubated at room temperature for 30 min. Clumps were broken up by pipetting up and down several times. The OMP were pelleted at 60 000 *g* for 1 h and washed once in 20 mM Tris (pH 7.2), and the protein concentration was determined by the method of Bradford [35]. The OMP were separated by 12% SDS-PAGE, later the proteins were transferred to a nitrocellulose membrane, at 300 mA for 1 h, and the membrane was blocked with TBS-Tween at 0.05 and 4% BSA. The membrane was washed with TBS and incubated with the following compounds coupled to horseradish peroxidase (HRP): 1 μg/mL of BapoLf, BholoLf, or lactoferricin B (fragment 4–14). Also, a competition binding test was made by incubating the membrane with BapoLf 100× (without HRP) and then incubating with each one of the proteins or the peptide coupled to HRP. All the membranes were revealed by chemiluminescence.

### Two-dimensional (2-D) SDS-PAGE

The outer membrane proteins were cleaned using Ready Prep™ 2-D cleanup kit (Bio-Rad), and proteins were dissolved in Ready Prep™ 2-D starter kit Rehydration/sample buffer (Bio-Rad); this sample was used to passively rehydrate the immobilized pH gradient (IPG) ReadyStrips (7 and 17 cm, linear, pH 3–10; Bio-Rad) during 16 h at 20 °C. Isoelectrofocusing (IEF) of the proteins was run in a Protean IEF Cell System (Bio-Rad) in the following steps: 250 V for a 20 min linear ramp, 250 V for a 1 h rapid ramp, 500 V for a 1 h rapid ramp, 400 V for a 2 h linear ramp, and 4000 V with a rapid ramp up to 10 000 V-h. After reduction and alkylation in the equilibration buffer, IPG strips were subjected to separation by MW on 12% SDS-PAGE. The gel was transferred onto a nitrocellulose membrane and processed for overlay with the different proteins labeled with HRP (see above). To identify the spots corresponding to the proteins that bind BLf, the image obtained from the revealed membrane and the gel stained with Coomassie blue G-250 (Bio-safe, Bio-Rad) were merged, using image J software [36] by aligning MW markers and membrane borders. Obtaining OMP that bound BapoLf as well as overlay assays were performed in three independent experiments.

### Identification of the bovine apolactoferrin binding proteins

The previously identified spots were cut from the stained Coomassie gel, and distained with ACN: 50 mM NH_4_HCO_3_ (1:1, v/v), and protein digestion was performed for 18 h at 37 °C with trypsin (masses grade, Promega V528A). The peptides were extracted from the digestion (ACN:H_2_O:formic acid 50:45:5 v/v), and the sample volume was decreased in an Eppendorf concentrator (Eppendorf 5301) and desalted using a C18 column (ZipTipC18). The sample was placed by sixfolds on the plate using α-cyan as a matrix, and analyzed in Maldi TOF/TOF 4800. Identification of the spots was made two times in independent samples. The MS/MS spectra data was searched in the database (NCBI-nr) and protein identification was performed using the MASCOT search algorithm (Version 1.6b9, Matrix Science) [37].

### Bioinformatics analyses

All the similarity searches of protein sequences were analyzed by BLAST [38]. Multiple sequence alignments were conducted using the Clustal Omega software website [39], the prediction of secondary structure was performed with PSS PRED [40], and edited using ESPRIPT 3.0 [41]. Search for protein families and predicting domains was performed at InterPro database at the European Bioinformatics Institute [42]. For a discrimination of the β-barrel structure of OMP and 2-D representation, MCMBB online tools were used [43]; the predicting and discriminating β-barrel OMP were performed with Hidden Markov Models PRED-TMBB [44]. Structure prediction was made using the I-TASSER server for protein 3-D models [45]. For quality estimation for 3-D models, a QMEAN server was used [46]. Molecular docking between *M. haemolytica* OMP and Lf was executed with the ClusPro server [47], and molecular graphics were performed with the USCF Chimera package; Chimera was developed by the Resource for Biocomputing, Visualization, and Informatics at the University of California, San Francisco (supported by NIGMS P41-GM103311) [48].

## Results

### Bovine hololactoferrin was not used as a sole iron source by *M. haemolytica*, and bovine apolactoferrin showed a bactericidal effect against this bacterium

**Figure 1:**
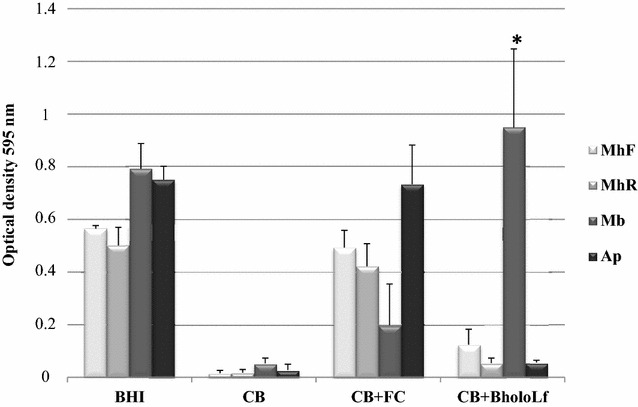
**Evaluation of bovine holo-lactoferrin (BholoLf) as a sole iron source in** ***Mannheimia haemolytica*** **strains (MhF and MhR).** *Moraxella bovis* was used as positive control and *Actinobacillus pleuropneumoniae* as a negative control of use of BholoLf. The bacterial growth was determined by OD_595nm_, at 24 h of incubation at 37 °C, in agitation (200 rpm) in different conditions: BHI, Brain heart infusion broth; CB, BHI chelated with dipyridyl; CB + FC, chelated BHI plus ferric chloride [80 μM]; CB + BholoLf, chelated BHI plus BholoLf [80 μM of iron]. The results are shown as the mean ± SD, *p < 0.05 versus CB and CB + BholoLf.

In iron-chelated BHI medium (Additional file 1), both strains of *M. haemolytica* required 80 μM iron (from FeCl_3_) for growing, under the conditions tested in this work; however, this bacterial species was not able to grow when BholoLf was added as a sole iron source to the iron-chelated BHI broth, even with 80 μM iron derived from BholoLf (Figure 1). The negative control *A. pleuropneumoniae* showed a similar negative behavior, as demonstrated in previous work [28]. *M. bovis*, the positive control of BholoLf utilization as an iron source, grew in the presence of this iron-charged protein. On the contrary, BapoLf inhibited the growth in vitro of both *M. haemolytica* strains; MIC obtained were 4.88 ± 1.88 and 7.31 ± 1.62 μM for MhF and MhR, respectively.

### Bovine lactoferrin mainly binds to two *M. haemolytica* outer membrane proteins

**Figure 2:**
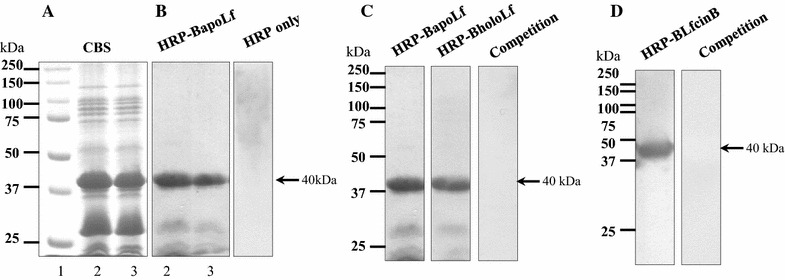
**Bovine lactoferrin (BapoLf and BholoLf) binding to** ***M. haemolytica*** **OMP. A** 12% SDS-PAGE of *M. haemolytica* OMP extracted with sarcosyl, stained with Coomassie blue. **B** OMP overlay, incubated with BapoLf coupled to horseradish peroxidase (HRP-BapoLf), the arrow shows the 40 kDa BapoLf binding protein; Lane 2, MhF (field strain); lane 3, MhR (reference strain). **C** Competence between BapoLf and HRP-BholoLf (MhF), OMP transferred were incubated with BapoLf and afterwards with HRP-BholoLf, the arrow shows the 40 kDa BLf binding protein. **D** OMP overlay (MhF), the membrane transferred was incubated with HRP-lactoferricin B (HRP-LfcinB), in the right line the membrane was incubated with BapoLf (without HRP) and afterwards with HRP-LfcinB; the arrow shows the 40 kDa BLfcin binding protein.

To determine whether *M. haemolytica* possesses BLf binding proteins, overlays were performed with extracted OMP and binding to HRP-coupled BholoLf and BapoLf was explored. A main band of 40 kDa was observed in the two strains for both forms of Lf (Figures 2A and B). To know whether this band could correspond to the same binding protein for BholoLf and BapoLf, a competition binding assay was made. As no band was visualized using BapoLf in competence with HRP-BholoLf, and vice versa, the result suggests that apo and holo forms of BLf bind to the same *M. haemolytica* OMP (Figure 2C). Next, a competition assay using HRP-BLfcin (BLf_4–14_ peptide) was made and no band was visualized using BapoLf in competence with HRP-BLfcin (Figure 2D). The results together suggest that *M. haemolytica* possesses at least one OMP that binds both forms of BLf and the binding could be through the N-terminus of BLf.

**Figure 3:**
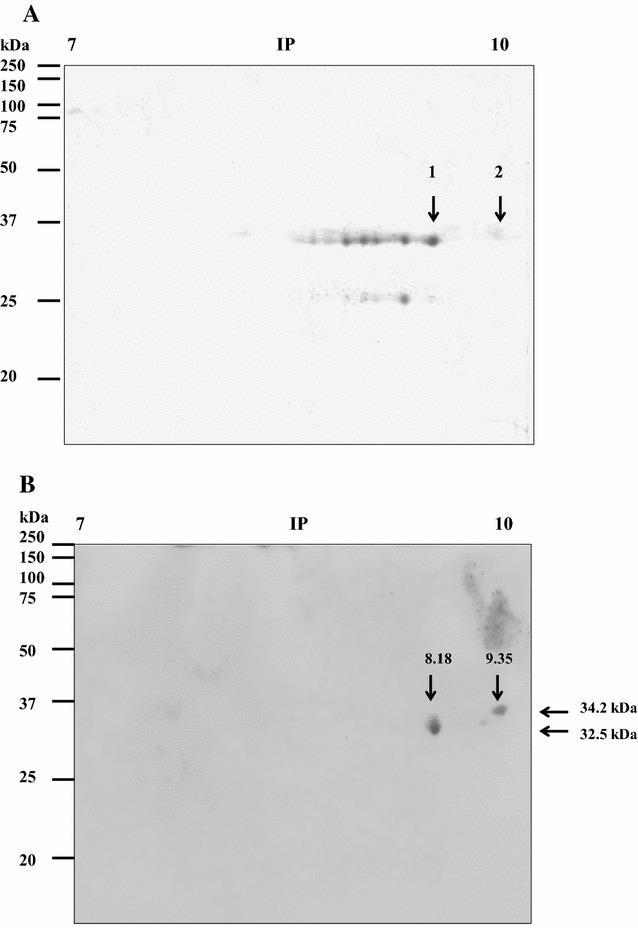
**Separation of the** ***M. haemolytica*** **outer membrane proteins that bind to bovine apolactoferrin (BapoLf) using 2-D gel electrophoresis. A** 2-D gel electrophoresis of the *M. haemolytica* OMP, stained with Coomassie blue. **B** Overlay of *M. haemolytica* OMP, from 2-D gel electrophoresis; the nitrocellulose membrane was incubated with HRP-BapoLf. The arrows show the spots of BapoLf binding proteins (**A**) and their MW and estimated IP (**B**), which were further identified by mass spectrometry.

**Figure 4:**
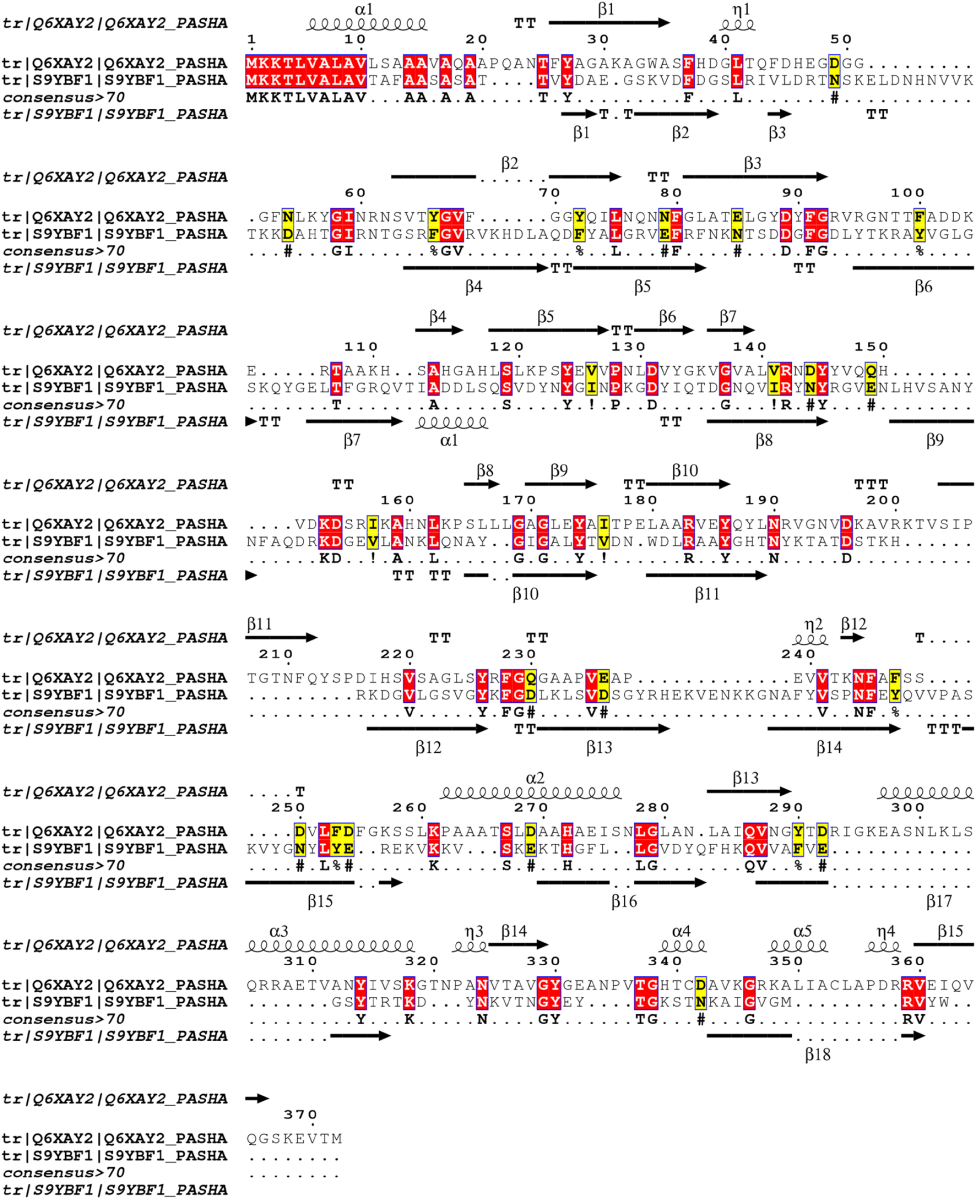
**Alignment between MhHM [UniProtKB: Q6XAY2] and MhMP sequences [UniProtKB: S9YBF1].** The prediction of the secondary structure performed with PSS PRED is shown in the top and the bottom of each sequence; the arrows show the β-sheets and the spiral α-helix structure. The red boxes show amino acid identity and the yellow boxes similarity [40, 41].

As the results with both strains of *M. haemolytica* were similar in the binding to BLf, only the MhF strain OMP were used to perform 2-D electrophoresis. The OMP separated by 2-D electrophoresis were transferred to a membrane and incubated with HRP-BapoLf. Interestingly, two spots of 32.9 and 34.2 kDa were found, with estimated IP of 8.18 and 9.35, respectively (Figure 3B). The corresponding two spots in the 2-D gel (Figure 3A) were cut and analyzed by Maldi-tof. A search was realized using the Mascot database of the MS/MS spectra obtained from two independent experiments. For both experiments, the two spots were identified as the heat-modifiable OMP (MhHM) [UniProt: Q6XAY2; gi|45758055] and an unknown OMP (MhMP) [UniProt: S9YBF1; gi|544866807] of *M. haemolytica*, with 34 and 39% coverage for the spots 1 and 2, respectively. Characteristics of the identified proteins are shown in Table 1. MhHM is encoded by the *ompA* gene, whereas the L278_12700 gene encodes MhMP. Both proteins have a signal peptide in the 1–19 position, and the analysis of peptides is listed in Additional files 2 and 3. Identity with other protein sequences was searched with NCBI-BLAST; the main identities are shown in Tables 2 and 3; in both cases the highest percentage of identity corresponds to proteins of the *Pasteurellaceae* family members. Sequences of MhHM and MhMP were aligned with Clustal Omega. Several identity sites were found, the alignment and prediction of secondary structure is shown in Figure 4. The differences in the sequences demonstrate that they are two different proteins of *M. haemolytica*; nevertheless, the identity regions could be the BLf binding sites in both OMP.

**Table 1:** **Characteristics of the** ***M. haemolytica*** **outer membrane proteins identified by Maldi-Tof**

Spot	Assigned names	Accession number	Amino acids	Experimental Mr(kDa)/IP	Gene	Signal peptide	Chain
1	Heat modifiable outer membrane protein (MhHM)	Q6XAY2 gi|45758055	372	32.9/8.18	*ompA*	1–19 position	20–372 position
2	Membrane protein (MhMP)	S9YBF1 gi|544866807	353	34.2/9.35	L278_12700	1–19 position	20–353 position

**Table 2:** **Identity of MhHM with related protein sequences** (The data were obtained from the Blast program [38])

Accession number	Protein	Max score	Query cover (%)	Identity (%)
AAO85792.1	Heat-modifiable OMP (*Mannheimia glucosida*)	666	100	95
WP_005818116.1	OMP P5 precursor (OMP P5) (*Actinobacillus minor*)	556	95	82
WP_025218170.1	Membrane protein (*Mannheimia varigena*)	555	100	83
WP_040218887.1	Membrane protein (*Haemophilus parahaemolyticus*)	549	100	78
WP_039198498.1	Membrane protein (*Actinobacillus equuli*)	540	100	75

**Table 3:** **Identity of MhMP with related protein sequences** (The data were obtained from the Blast program [38])

Accession number	Protein	Max score	Query cover (%)	Identity (%)
AGI35167.1	OMP P2-like protein (*Mannheimia haemolytica* USDA-ARS-USMARC-185)	638	100	91
WP_025217393.1	Membrane protein (*Mannheimia varigena*)	506	100	91
AHG75679.1	OMP P2-like protein (*Mannheimia varigena* USDA-ARS-USMARC-1296)	506	100	71
WP_014991497.1	Membrane protein (*Actinobacillus suis*)	441	93	64
WP_009874692.1	Membrane protein *(Actinobacillus pleuropneumoniae*)	427	100	61

### MhHM and MhMP domains

Protein domains were searched in the website InterPro. MhHM belongs to the protein family OmpA [IPR002368]; proteins of this family contain two domains; one of them is the OmpA-like transmembrane domain at the N-terminus [IPR000498] consisting of an eight stranded β-barrel and the other one is an OmpA C-like domain [IPR006665], similar to the C-terminal domain of OmpA peptidoglycan-binding domain. Concerning the other BLf binding protein, MhMP, the domain searched in website InterPro resulted in a conserved domain in Gram-negative porins [IPR023614].

### Both MhHM and MhMP are transmembrane proteins

**Figure 5:**
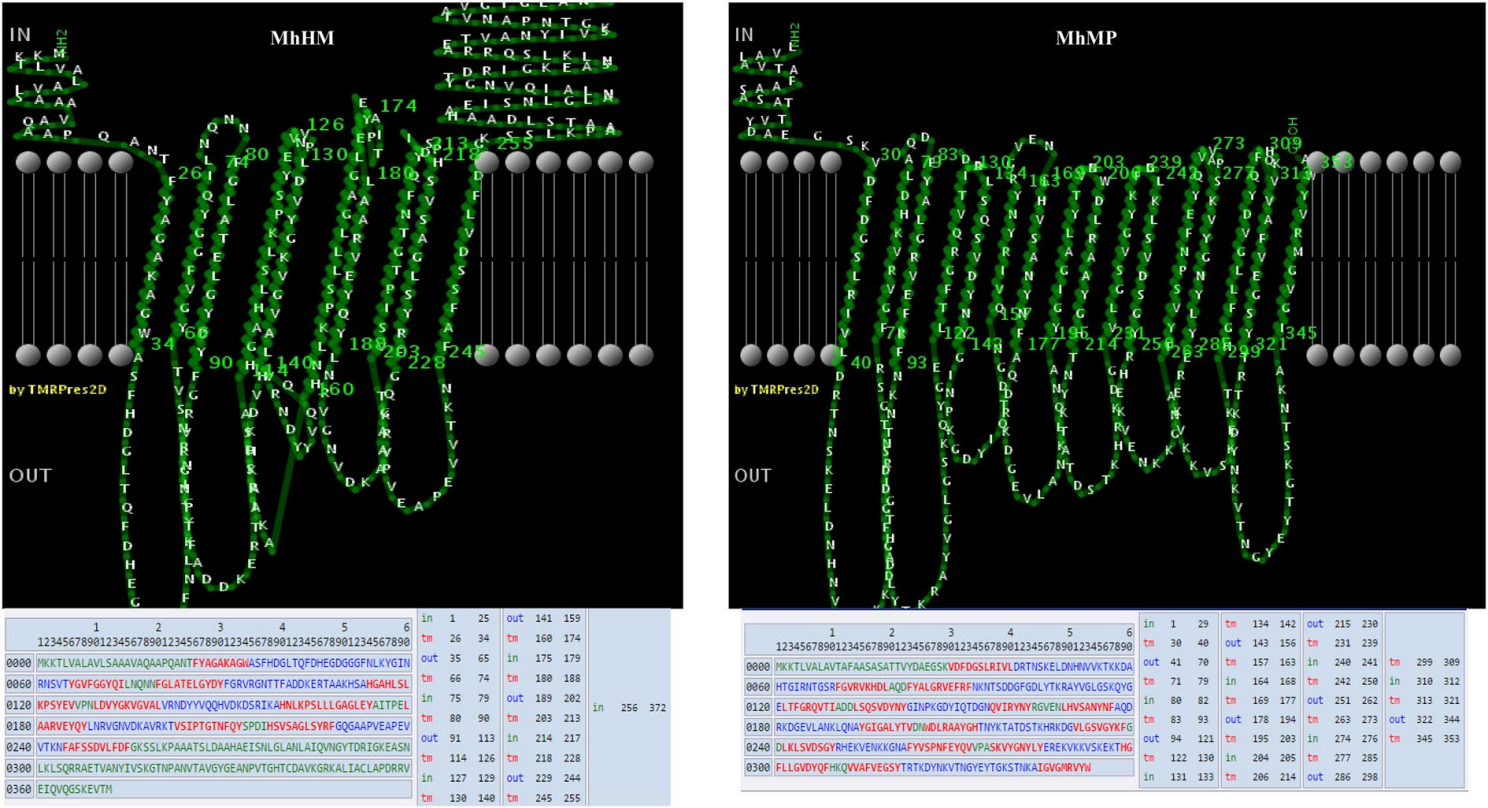
**Prediction in Pred-TMBB of the protein localization at the membrane of MhHM and MhMP.** In the sequence, amino acids in green represent the inner localization; amino acids in red, the transmembrane localization; amino acids in blue, the outside localization [44].

MhHM and MhMP sequences were analyzed with MCMBB. The scores obtained were 0.028 for MhMH and 0.046 for MhMP; where a score greater than zero, indicates that the protein is more likely to be a β-barrel OMP. The analysis with Pred-TMBB confirmed the transmembrane localization of the proteins. Figure 5 shows how the amino acid sequence could be organized at the OM.

### Prediction of MhHM and MhMP 3-D model

**Figure 6:**
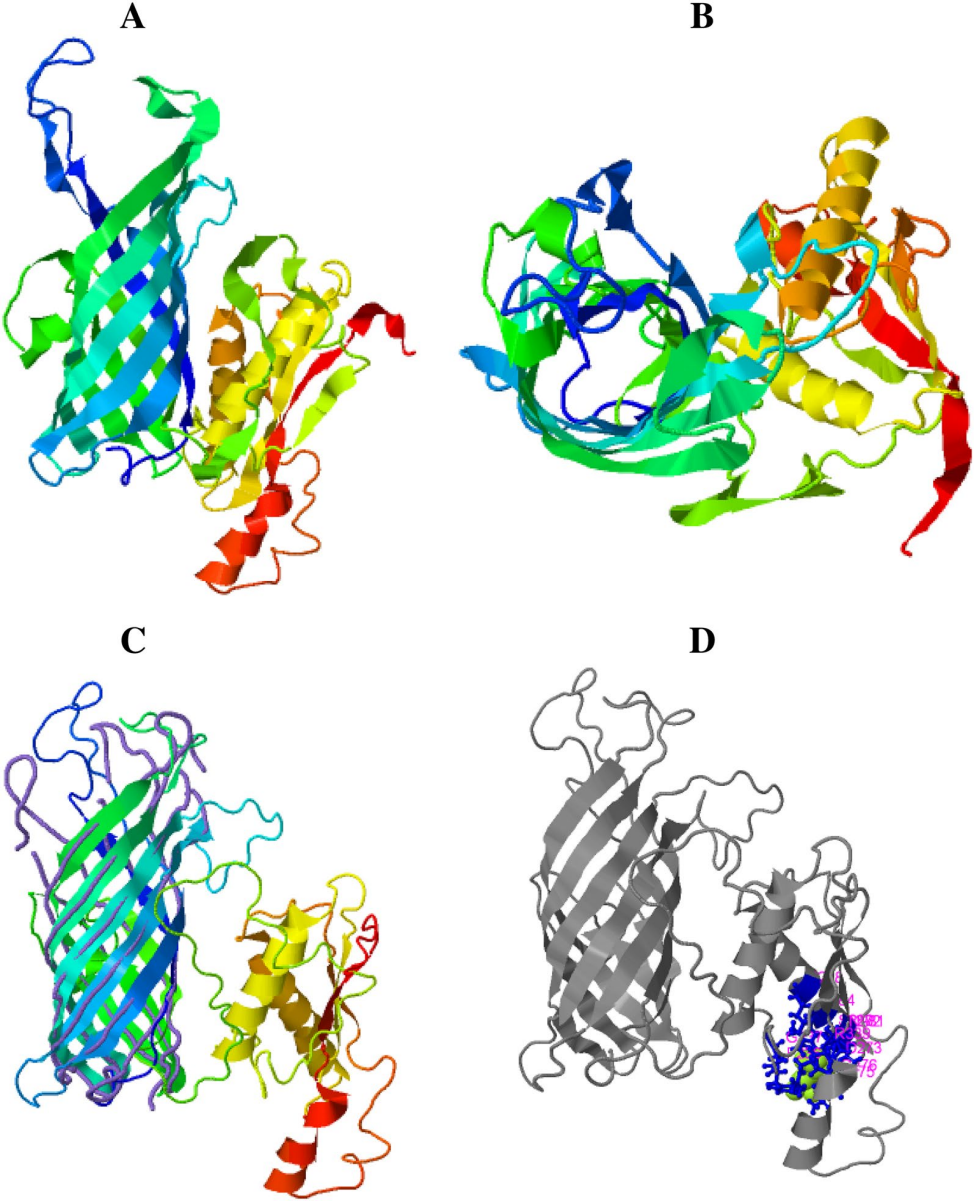
**3-D model for MhHM in I-Tasser server with a C-score of −3.56. A** Lateral view in rainbow ribbon diagram. **B** Top view of the rainbow ribbon diagram. **C** Alignment between the close structure OmpA transmembrane domain of *E. coli,* which is represented by purple lines, and MhHM, in the rainbow ribbon diagram. **D** Prediction of the ligand binding site; the consensus binding residues of OmpA-like domain of *A. baumannii* are represented by blue sticks and MhHM in gray color diagram (before as rainbow ribbon).

A predicted 3-D model for MhHM and MhMP was computed using the I-TASSER server. This analysis generated five top models; in the case of MhHM, the model number 3 was selected because it coincides with the structure of the MhHM protein domains, this model has a C-score of −3.56 (Figure 6A). C-score is typically in the range of −5 to 2, where a C-score of a higher value signifies a model with a higher confidence. A movie file shows in more detail the model of MhHM (Additional file 4). In the top ten threading templates used by I-Tasser, the OmpA of *E. coli* [PDB: 3nb3A] has the highest norm. Z-score (4.17), norm. Z-score is the normalized Z-score of the threading alignments; alignment with a normalized Z-score >1 means a good alignment and vice versa. Furthermore, the server COFACTOR of I-Tasser provides proteins with a highly similar structure; and often these proteins have similar function due to the structure similarity. For MhHM, the ranking 1 protein was the OmpA transmembrane domain of *E. coli* [PDB: 1BXW], with a TM-score of 0.404 (metric for measuring the structural similarity of two protein models) (Figure 6B). About the prediction of the ligand binding site, the OmpA-like domain from *Acinetobacter baumannii* [PDB: 3TD4] was the ranking 1 protein, with a C-score of 0.08 and consensus binding residues Glu236, Ala237, His272, Ala273, Ile275, Ser276, Leu280, Ala281, Ala284, Asn288, and His339; this OmpA-like domain has been crystallized and the binding to peptidoglycan has been reported (Figure 6C) [49].

**Figure 7:**
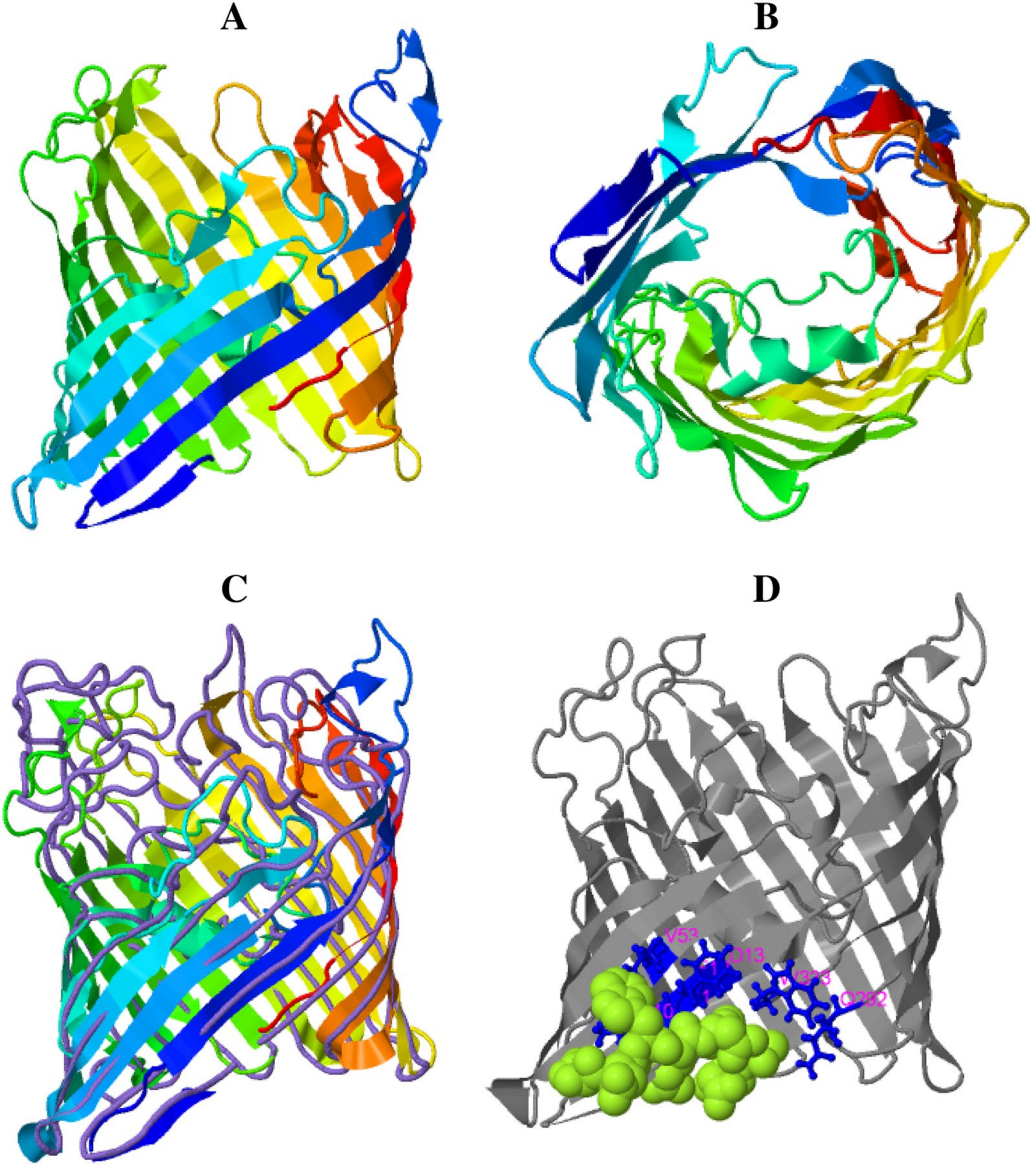
**3-D model for MhMP in I-Tasser server with a C-score of 1.02. A** Lateral view in rainbow ribbon diagram. **B** Top view of the rainbow ribbon diagram. **C** Alignment between the close structure OmpC of *E. coli,* which is represented by purple lines, and MhMP by the rainbow ribbon diagram. **D** Prediction of ligand binding site; the consensus binding residues of Omp 38 of *Delftia acidovorans* are represented by blue sticks and the ligand malate by green spheres. MhMP in gray color diagram (before as rainbow ribbon).

For MhMP, the top ten threading templates used by I-Tasser included the OmpF of *E. coli* [PDB: 1HXT] with Z-score of 4.0 and Omp32 of *Delftia acidovorans* [PDB: 2FGR] with a norm. Z-score of 4.35. The model of the 3-D structure for MhMP is shown in Figure 7A (C-score = 1.02). An additional movie file shows the model of MhMP in more detail (Additional file 5). The Omp32 of *D. acidovorans* (TM-score = 0.914), OmpC [PDB: 2XE1] (Figure 7B), and OmpF proteins of *E. coli* have the closest structural similarity to MhMP, with a TM-score of 0.847 and 0.843, respectively. On the contrary, the prediction of the ligand binding site was the *Delftia acidovorans* Omp32; with a C-score of 0.03 and ligand binding sites residues Val10, Tyr11, Ala12, Phe13, Val53, Lys292, and Gly333. This Omp32 has calcium and sulfate ligands [BioLip: BL0089114] (Figure 7C) [50].

The quality estimation of 3-D models was calculated with QMEAN for both proteins (MhHM score = 0.415 and MhMP score = 0.319). For the two proteins, a sequence without signal peptide was considered to generate the 3-D model; this because it produces a better I-Tasser C-score and QMEAN score, although the QMEAN score resulted low in both cases. This was expected because they have a β-barrel structure and are transmembrane proteins [51].

### Molecular docking between *M. haemolytica* MhHM and MhMP OMP and bovine lactoferrin

**Figure 8:**
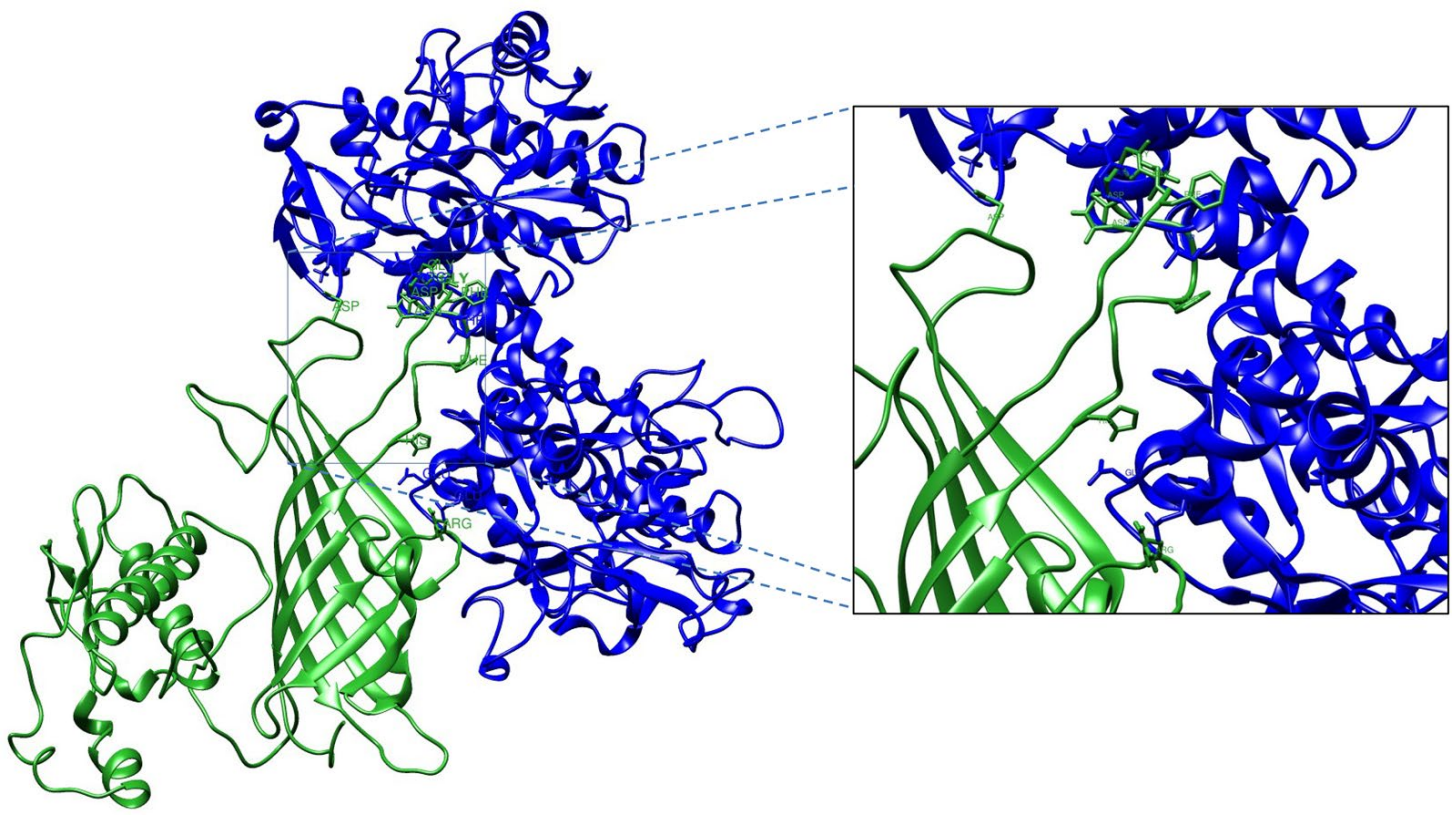
**Molecular docking between MhHM model (green ribbon diagram) and bovine lactoferrin (blue ribbon diagram).** The residues in sticks representation show the possible amino acids involved in the docking between the two proteins [47].

The protein docking was predicted with the ClusPro server. The docking models between *M. haemolytica* OMP and BLf were chosen based on the predictions made in TMBB about the amino acids located outside of the bacterial membrane. Figure 8 shows the docking for MhHM and BholoLf [PDB: 1BLF] (BholoLf form is the only one available in the protein databank). The amino acids of MhHM involved in the docking are His38, Phe44, Asp49, Gly50, Gly51, Gly52, Asn54, Asp152, and Arg 191, corresponding to the outside predicted with Pred-TMBB and the BLf amino acids are Lys243, Glu659, Glu664, Thr688, and Arg689; the Lys243 residue is located in the N-terminus and the other residues in C-terminus [52].

**Figure 9:**
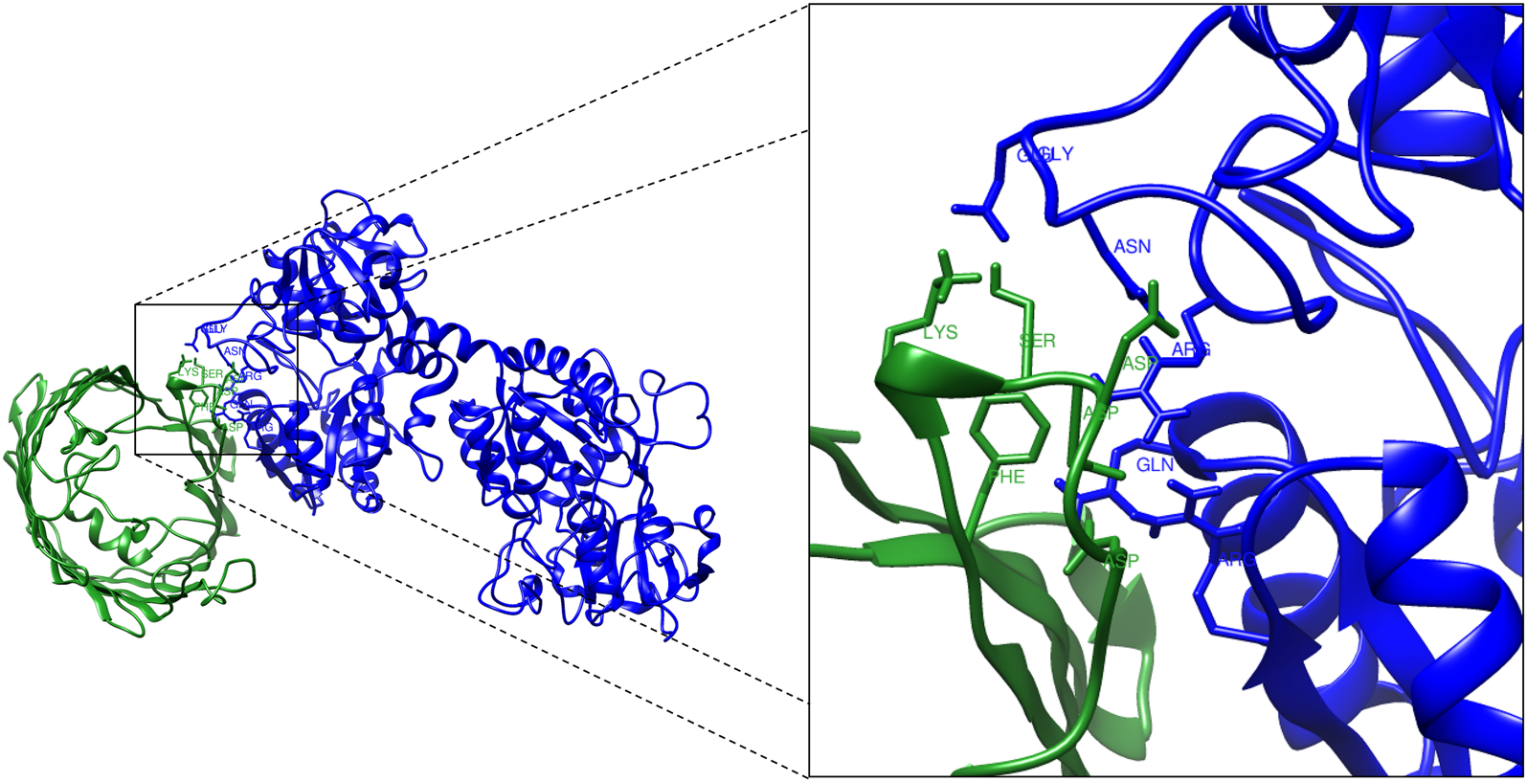
**Molecular docking between MhMP model (green ribbon diagram) and bovine lactoferrin (blue ribbon diagram).** The residues in sticks representation show the possible amino acids involved in the docking between the two proteins [47].

The docking between MhMP and BholoLf is represented in Figure 9. In this case, the residues involved are Phe93, Lys95, Ser98, Asp99, Asp100, and Asp104 of the MhMP, and Gln13, Arg38, Glu176, Gly177, Asn179, and Arg186 for BholoLf corresponding to the N-terminus, responsible for the bactericidal effect. Additional movie files show in more detail the docking between the proteins (Additional files 6 and 7).

## Discussion

Our results point out that *M. haemolytica* is not able to use BholoLf as a sole iron source for growth. Ogunnariwo and Schryvers [18] looked for the *lbpA* and *lbpB* sequences in several members of the *Pasteurellaceae* family by PCR; these genes encode for proteins LbpA and LbpB, which are OMP that allow *Neisseriaceae* family bacteria to take up the iron from holoLf. As they found no amplification of these sequences in *M. haemolytica*, our results complement those from these authors; however, we cannot discard the binding of *M. haemolytica* to BholoLf through an alternative mechanism to the known Lbps.

BapoLf showed a bactericidal effect for *M. haemolytica*. In other members of the *Pasteurellaceae* family, the bactericidal effect of apoLf has also been described; our group reported a higher MIC (11.78 μM) for BapoLf in the pig pathogen *A. pleuropneumoniae* serotype 1, than the MIC (4.88 ± 1.88 and 7.31 ± 1.62 μM) obtained in *M. haemolytica* in this work [28]. In other studies, a lower concentration of HapoLf (3.8 μM) was found for killing the human oral pathogen *A. actinomycetemcomitans* [53]. Therefore, the MIC we obtained for BapoLf in *M. haemolytica* are within the range of those for other species [54]. As BapoLf was bactericidal in vitro for *M. haemolytica*, it also could have a similar effect in this species in vivo and could be used against bovine Mannheimiosis. The bactericidal effect of BLf could be due to the binding of cationic BapoLf to the anionic *M. haemolytica* surface, and/or to the interaction with MhHM and MhMP proteins, in both cases leading to an OM destabilization, as occurs in other bacteria [24].

The IEF assay shows that *M. haemolytica* possesses two BLf binding proteins, one of 32.9 kDa and another one of 34.2 kDa with estimated IP of 8.18 and 9.35, respectively. The MW were different from that observed in 1-D (40 kDa), perhaps due to the boiling sample preparation in 1-D and the heat-modifiable property of the OmpA (see below) [55]. By Maldi TOF/TOF 4800 analysis, the proteins corresponded to the heat modifiable OMP (MhHM) and an unknown OMP (MhMP) of *M. haemolytica*, respectively. MhHM is encoded by the *ompA* gene, whereas L278_12700 gene encodes MhMP. Sequences of MhHM and MhMP were aligned with Clustal Omega, a multiple sequence alignment program that uses seeded guide trees and HMM profile–profile techniques to generate alignments between sequences. The differences in the sequences demonstrate that they are two different proteins of *M. haemolytica*; nevertheless, the identity regions could be the BLf binding sites in both OMP. Apparently, the site of BLf interaction with *M. haemolytica* OMP is the N-terminus, since BLfcin (BLf 4–14 peptide) avoids the binding with BapoLf in the competition binding assays; and HRP-BLfcin also binds to the 40 kDa band. Our results showing that the same OMP can bind BapoLf and BholoLf, agree with those for HapoLf and HholoLf binding to OmpC and OmpF in enteropathogenic *Escherichia coli* (EPEC) [26]. As BapoLf and BholoLf are bound to the same *M. haemolytica* OMP, thus the function of these Lf binding proteins in vivo could be explained in two ways: BapoLf binding to *M. haemolytica* OMP causes bacterial death and then the binding might help to avoid the infection process. As *M. haemolytica* Lf binding proteins cannot discriminate between the iron content of BLf, thus the binding site to BLf could not be related with iron binding. On the contrary, we found that *M. haemolytica* did not use BholoLf as an iron source, thus we can speculate that this iron-charged protein could be participating in the inhibition of some pathogenicity mechanisms in this bacterial species, such as occurs in the biofilm production by *A. pleuropneumoniae* [28]. In this context, BholoLf reduced the adhesion of enterotoxigenic *E. coli* (ETEC) to the JTC17 cell line and different portions of mouse intestine [56]. In addition, [57] reported that BholoLf (30% iron saturation) suppressed the adhesion of EPEC to HEp-2 cells by 88%. Other experiments are necessary to demonstrate the role that MhHM (OmpA) and MhMP (porin) play in *M. haemolytica* in their interaction with BholoLf.

Protein domains were searched in the website InterPro, which is a resource that provides functional analysis of protein sequences by classifying them into families and predicting the presence of domains and important sites. MhHM possesses two domains, OmpA-like and OmpA-C like. Well-studied OmpA domains containing proteins in bacteria include OmpA, lipoprotein PAL, motor protein MotB in *E. coli*, RmpM of *Neisseria meningitidis* that interacts with other components of the OM, and the *Vibrio alginolyticus* flagellar motor proteins PomB and MotY that interact with the inner membrane [58]. The OmpA protein is an integral component of the OM and is highly conserved in Gram negative bacteria. The protein has characteristic heat-modifiable properties [59], is present in a high copy number (>10^5^/cell), and is immunogenic [60]. Functions that have been attributed to OmpA include the maintenance of OM integrity and cell shape. OmpA also has other roles, for example, it acts as a bacteriophage receptor, takes part in conjugation, and confers resistance to the bactericidal effect of the serum [61]. In *M. haemolytica* the *ompA* gene has been cloned and sequenced and the immunological properties of OmpA have been investigated [11]. In addition, OmpA fibronectin-binding activity has been demonstrated [62], as well as the binding to bovine bronchial epithelial cells [12]. Therefore, the binding of BapoLf to MhHM (OmpA) could alter the OM integrity and cause bacterial death; additionally it may affect binding to fibronectin and epithelial cells, which possibly decreases bacterial adherence. In accordance with that, in other pathogens like *E. coli* and *Shigella dysenteriae*, the inhibition caused by Lf in bacterial adherence has been reported [63, 64].

Concerning MhMP, this OMP possesses the conserved Gram-negative porin domain; the porins form ion selective channels for small hydrophilic molecules. X-ray structured analyses of several bacterial porins have revealed a large 16 stranded anti-parallel structure enclosing the transmembrane pore. Trimers are stabilized by hydrophilic clamping of loop L2 [65]. Currently, the function of MhMP is unknown.

MhHM and MhMP sequences were analyzed with MCMBB, an algorithm that achieves high accuracy in discriminating β-barrel OMP from globular and alpha-helical membrane proteins. The model allows a correct classification rate of 90.08% for β-barrel proteins and of 92.67% for globular proteins. When submitting alpha-helical membrane proteins to analysis, the method shows 100% accuracy. Also, the analysis with Pred-TMBB, a method based on a Hidden Markov Model, capable of predicting the transmembrane beta-strands of the Gram-negative bacteria OMP, and of discriminating such proteins from water-soluble ones when screening large datasets, confirms the transmembrane localization of the proteins, and how the amino acid sequences could been organized at the OM. These in silico analyses strongly suggest that the structures of MhHM and MhMP are β-barrel and transmembrane proteins.

By using the I-TASSER server, MhHM protein possesses a similar structure to the OmpA transmembrane domain of *E. coli*. The ligand binding site was similar to the OmpA-like domain from *Acinetobacter baumannii*; this OmpA-like domain has been crystallized and the binding to peptidoglycan has been reported [49]. MhMP has a porin structure, and a correlation between Lf binding to porins and the Lf-mediated antimicrobial bactericidal effect has been reported in other bacterial species [66]. Erdei et al. [26] presented evidence for Lf interaction with porins OmpF and OmpC. These porins bind to HapoLf and HholoLf in *E. coli* which seems to be accountable for the antimicrobial effect of Lf in *E. coli*; these data fully agree with the results found in the present work, since BapoLf has a bactericidal effect in *M. haemolytica* and binds to the putative porin MhMP, structurally close to OmpF and OmpC.

The protein docking was predicted with the ClusPro server. The docking algorithms evaluate billions of putative complexes, retaining a preset number with favorable surface complementarities. A filtering method is then applied to this set of structures, selecting those with good electrostatic and desolvation free energies for further clustering. The program output is a short list of putative complexes ranked according to their clustering properties [67]. The docking residues of MhMP are localized in loop 2, which is the site of interaction to form the homotrimer. Possibly, the BLf binding could affect the homotrimer conformation and in consequence produce a membrane destabilization. Interestingly, the BLf is predicted to bind to different sites at the *M. haemolytica* Omps, the C-terminus within MhHM and the N-terminus within MhMP, and in different residues of the Omps. Because the models are approximations of how the proteins could be binding, and since other components found in the OM are not considered, this study provides only a guide of the possible binding sites and further studies are needed to confirm these results.

In conclusion, this work sheds light on the relationship between *M. haemolytica* and BLf: it was determined that *M. haemolytica* does not use BholoLf as a sole iron source, and that BapoLf displays a bactericidal effect on *M. haemolytica*. Both forms of the protein bind to proteins MhHM and MhMP. This interaction could be responsible for the bactericidal effect of BapoLf on *M. haemolytica*, in agreement to what happens in other Gram negative bacteria. The results suggest that BapoLf could be added to the bovine mannheimiosis treatment to help eliminate the infection process in vivo.

## Additional files

10.1186/s13567-016-0378-1 ***Mannheimia haemolytica*** **growth in different concentrations of the chelating agent 2′2 dipyridyl.** *M. haemolytica* strain F (field isolate) and strain R (reference strain) were grown in BHI broth with 2,2’-dipyridyl, and OD (595 nm) was recorded at 0 and 18 h of incubation at 37 °C in agitation (200 rpm).
10.1186/s13567-016-0378-1 **List of peptides obtained by Maldi-Tof/Tof to identify the spot 1.** MhHM score 125, Protein score is −10*Log(P), where *P* is the probability that the observed match is a random event. Protein scores greater than 86 are significant (*p* < 0.05). Protein scores greater than 86 are significant (*p* < 0.05). Protein sequence coverage 34%.
10.1186/s13567-016-0378-1 **List of peptides obtained by Maldi-Tof/Tof to identify the spot 2.** MhMP score 124, Protein score is −10*Log(P), where *P* is the probability that the observed match is a random event. Protein scores greater than 86 are significant (*p* < 0.05). Protein scores greater than 86 are significant (*p* < 0.05). Protein sequence coverage 39%.
10.1186/s13567-016-0378-1 **Video of the MhHM protein 3-D model.** This video shows the 3-D structure of MhHM obtained by I-Tasser server (cartoon representation). Domain OmpA and domain OmpA-C like are observed in blue-green and yellow-orange colors, respectively.
10.1186/s13567-016-0378-1 **Video of the MhMP protein 3-D model.** This video shows the 3-D structure of MhMP obtained by by I-Tasser server and visualized with Chimera (cartoon representation). Porin domain is observed in rainbow colors.
10.1186/s13567-016-0378-1 **Video of the molecular docking between MhHM and BLf.** This video shows the docking obtained by ClusPro server and visualized with Chimera (cartoon representation). MhHM is observed in green and bovine lactoferrin in blue. The residues in sticks representation show the possible binding between the two proteins.
10.1186/s13567-016-0378-1 **Video of the molecular docking between MhMP and BLf.** This video shows the docking obtained by ClusPro server and visualized with Chimera (cartoon representation). MhMP is observed in green and bovine lactoferrin in blue. The residues in sticks representation show the possible binding between the two proteins.

## Abbreviations

HapoLf: human apolactoferrin
HholoLf: human hololactoferrin
Lf: lactoferrin
BapoLf: bovine apolactoferrin
BholoLf: bovine hololactoferrin
OMP: outer membrane proteins
MIC: minimal inhibitory concentration
MhF: *M. haemolytica* field strain
MhR: *M. haemolytica* reference strain
OM: outer membrane
BLfcin: bovine lactoferricin
IP: isoelectric point
IPG: immobilized pH gradient
IEF: isoelectrofocusing
EPEC: enteropathogenic *E. coli*
ETEC: enterotoxigenic *E. coli*
