# Supplementary material for: Two outer membrane proteins are bovine lactoferrin-binding proteins in Mannheimia haemolytica A1
Source: Vet Res. 2016 Sep 6;47(1):93. doi: 10.1186/s13567-016-0378-1 (PMC5013584; doi:10.1186/s13567-016-0378-1)
Supplement: Supplementary file 1 — 10.1186/s13567-016-0378-1 Mannheimia haemolytica growth in different concentrations of the chelating agent 2′2 dipyridyl. M. haemolytica strain F (field isolate) and strain R (reference strain) were grown in BHI broth with 2,2’-dipyridyl, and OD (595 nm) was recorded at 0 and 18 h of incubation at 37 °C in agitation (200 rpm). [file 13567_2016_378_MOESM1_ESM.docx]

**Additional Table 1**

*Mannheimia haemolytica* growth in different concentration of 2, 2’- dipyridyl.

| Time (h) | Strain | **2, 2’- dipyridyl concentration (mM)** | | | | | | | | | |
| --- | --- | --- | --- | --- | --- | --- | --- | --- | --- | --- | --- |
|  |  | **0** | **0.10** | **0.15** | **0.20** | **0.25** | **0.30** | **0.35** | **0.40** | **0.45** | **0.50** |
| 0 | F | 0.02 | 0.02 | 0.02 | 0.02 | 0.02 | 0.02 | 0.02 | 0.02 | 0.02 | 0.02 |
|  | R | 0.02 | 0.02 | 0.02 | 0.02 | 0.02 | 0.02 | 0.02 | 0.02 | 0.02 | 0.02 |
| 18 | F | 0.34 | 0.33 | 0.31 | 0.23 | 0.20 | 0.06 | 0.01 | 0.01 | 0.01 | 0.01 |
|  | R | 0.33 | 0.32 | 0.30 | 0.24 | 0.19 | 0.05 | 0.02 | 0.01 | 0.01 | 0.01 |

*M. haemolytica* strain F (field isolate) and strain R (reference strain) were grown in BHI broth with 2, 2’- dipyridyl, and OD (595 nm) was recorded at 0 and 18 h of incubation at 37 °C in agitation (200 rpm). The results are shown as the mean of two independent experiments each in triplicate.
