# Supplementary material for: Two outer membrane proteins are bovine lactoferrin-binding proteins in Mannheimia haemolytica A1
Source: Vet Res. 2016 Sep 6;47(1):93. doi: 10.1186/s13567-016-0378-1 (PMC5013584; doi:10.1186/s13567-016-0378-1)
Supplement: Supplementary file 2 — 10.1186/s13567-016-0378-1 List of peptides obtained by Maldi-Tof/Tof to identify the spot 1. MhHM score 125, Protein score is −10*Log(P), where P is the probability that the observed match is a random event. Protein scores greater than 86 are significant (p < 0.05). Protein scores greater than 86 are significant (p < 0.05). Protein sequence coverage 34%. [file 13567_2016_378_MOESM2_ESM.docx]

| **Peptide No.** | **Start-End** | **Observed** | **Mr(expt)** | **Mr(calc)** | **Peptide sequences** |
| --- | --- | --- | --- | --- | --- |
| 1 | 57-61 | 622.3480 | 621.3407 | 621.3234 | K.YGINR.N |
| 2 | 136-142 | 713.4510 | 712.4437 | 712.4596 | K.VGVALVR.N |
| 3 | 143-156 | 1766.8170 | 1765.8097 | 1765.8020 | R.NDYYVQQHVDKDSR.I |
| 4 | 157-183 | 2859.6420 | 2858.6347 | 2858.6381 | R.IKAHNLKPSLLLGAGLEYAITPELAAR.V |
| 5 | 159-183 | 2618.4630 | 2617.4557 | 2617.4591 | K.AHNLKPSLLLGAGLEYAITPELAAR.V |
| 6 | 184-191 | 1084.5040 | 1083.4967 | 1083.5349 | R.VEYQYLNR.V |
| 7 | 201-227 | 2925.5280 | 2924.5207 | 2924.4668 | R.KTVSIPTGTNFQYSPDIHSVSAGLSYR.F |
| 8 | 202-227 | 2797.4290 | 2796.4217 | 2796.3719 | K.TVSIPTGTNFQYSPDIHSVSAGLSYR.F |
| 9 | 307-318 | 1350.7140 | 1349.7067 | 1349.7303 | R.RAETVANYIVSK.G |
| 10 | 319-345 | 2700.3460 | 2699.3387 | 2699.2610 | K.GTNPANVTAVGYGEANPVTGHTCDAVK.G |

**Additional file 2 List of peptides obtained by Maldi-Tof/Tof to identify the spot 1.**
