# Supplementary material for: Two outer membrane proteins are bovine lactoferrin-binding proteins in Mannheimia haemolytica A1
Source: Vet Res. 2016 Sep 6;47(1):93. doi: 10.1186/s13567-016-0378-1 (PMC5013584; doi:10.1186/s13567-016-0378-1)
Supplement: Supplementary file 3 — 10.1186/s13567-016-0378-1 List of peptides obtained by Maldi-Tof/Tof to identify the spot 2. MhMP score 124, Protein score is −10*Log(P), where P is the probability that the observed match is a random event. Protein scores greater than 86 are significant (p < 0.05). Protein scores greater than 86 are significant (p < 0.05). Protein sequence coverage 39%. [file 13567_2016_378_MOESM3_ESM.docx]

**Additional file 3 List of peptides obtained by Maldi-Tof/Tof to identify the spot 2.**

| **Peptide No.** | **Start–End** | **Observed** | **Mr(expt)** | **Mr(calc)** | **Peptide sequence** |
| --- | --- | --- | --- | --- | --- |
| 1 | 38–55 | 2094.1740 | 2093.1667 | 2093.1229 | R.IVLDRTNSKELDNHNVVK.T |
| 2 | 59–74 | 1743.9350 | 1742.9277 | 1742.8925 | K.DAHTGIRNTGSRFGVR.V |
| 3 | 75–88 | 1632.8780 | 1631.8707 | 1631.8420 | R.VKHDLAQDFYALGR.V |
| 4 | 93–109 | 1977.9810 | 1976.9737 | 1976.9228 | R.FNKNTSDDGFGDLYTKR.A |
| 5 | 110–126 | 1845.9990 | 1844.9917 | 1844.9421 | R.AYVGLGSKQYGELTFGR.Q |
| 6 | 118–126 | 1070.5330 | 1069.5257 | 1069.5193 | K.QYGELTFGR.Q |
| 7 | 148–160 | 1478.7410 | 1477.7337 | 1477.7161 | K.GDYIQTDGNQVIR.Y |
| 8 | 148–164 | 2075.0620 | 2074.0547 | 2073.9868 | K.GDYIQTDGNQVIRYNYR.G |
| 9 | 165–181 | 1933.9540 | 1932.9467 | 1932.9078 | R.GVENLHVSANYNFAQDR.K |
| 10 | 165–182 | 2062.0590 | 2061.0517 | 2061.0028 | R.GVENLHVSANYNFAQDRK.D |
| 11 | 279–287 | 1176.5740 | 1175.5667 | 1175.5611 | K.VYGNYLYER.E |
| 12 | 312–323 | 1355.6970 | 1354.6897 | 1354.6881 | K.QVVAFVEGSYTR.T |
